# Semantic Scholar

**DOI:** 10.5195/jmla.2018.280

**Published:** 2018-01-02

**Authors:** Suzanne Fricke

## INTRODUCTION

Eagerly awaited by researchers for years, concrete examples of artificial intelligence–enabled search engines are beginning to emerge. Founded by the nonprofit Allen Institute for Artificial Intelligence (AI2), Semantic Scholar began as a search engine for computer science, geoscience, and neuroscience in 2015. In response to researchers’ inability to keep pace with reading all of the publications in their disciplines, the purpose of the project is automated learning from text in order to overcome information overload.

This project is just one by AI2 designed to fulfill the organization’s mission of “[artificial intelligence] for the common good” [1]. Semantic Scholar had ten million articles prior to branching into biomedical literature in 2017 [2, 3]. It is reviewed here as an artificial intelligence–based search engine now poised to play a large role in health disciplines. Semantic Scholar is notable among artificial intelligence search for the velocity with which it is being developed and the strength and knowledge of the development team.

Collaborators on the project include CiteSeerX, a product of the Colleges of Information Science and Technology at Pennsylvania State University [4]. CiteSeerX, preceded by CiteSeer, attempts to index full-text portable document format files (PDFs) in computer and information science using completely autonomous citation indexing (ACI).

## SEARCH RESULTS

Features of Semantic Scholar are in keeping with the mission to save the user time. By limiting results, Semantic Scholar renews a scientist’s joy of discovery once again. Searches that return tens of thousands of results in Google Scholar and thousands in PubMed return a few hundred in Semantic Scholar, all directly relevant. Semantic Scholar removes the long tail of search results, allowing one to quickly get up to speed on one’s disciplines, while limiting the distraction caused by less relevant research. Semantic Scholar also saves time by its drive to provide full-text and mobile-enabled design. It truly is a search engine designed by researchers for researchers.

## CITATION ANALYTICS

The citation analytics features graphically represent citation velocity and author influence scores that help researchers pre-assess quality, in much the same way that pre-assessed evidence levels aid clinicians. Displays quickly visualize those elements that researchers are most interested in—references and citations, methods as a limiter, and graphs and tables—without requiring a great deal of reading.

With a growing need for researchers and institutions to show impact, highly cited authors are emphasized with influence scores, highly influential citations [5], total citations, a citations-per-year graph, and a citation velocity score. Authors with fewer than 50 citations are merely noted as “<50” citations. Given sufficient citations, author maps indicate those most influenced by an author and those with the greatest influence on an author. The reference list brings deeper meaning to citations by showing where and how often a reference is cited in the paper through a display of the semantic context or contexts. Semantic Scholar attempts to combine conventional citation metrics and altmetrics with the “cited by” function seen elsewhere in Web of Science and Google Scholar, as well as links to tweets about citations.

## SCOPE

Given the origins of the search engine, neuroscience still predominates in the biomedical literature that Semantic Scholar indexes. The nature of searches may change as more citations are added.

## INTEROPERABILITY

Semantic Scholar does not offer an application programming interface (API), preventing interoperability with other health care or bibliometric systems. Users can copy and paste citations in Bibtex, Endnote, Modern Language Association (MLA), American Psychological Association (APA), and *Chicago Manual of Style* styles, but citations cannot be exported. Users can create reading lists if they sign in—via Facebook, Twitter, or Google—but those lists, also, cannot be exported. Authentication should be with the same account each time; that is, a sign-in with Facebook will not link to a sign-in with Twitter. Users cannot create alerts to notify them of new search results.

## SEARCH REFINEMENT

Favoring simplicity of interface, Semantic Scholar offers only a few options for refining and sorting search results. It sorts only by relevance and publication date. While it does allow truncation, it does not support Boolean or phrase searching. Some limiters found here are not found in other databases—such as Data Set Used, Cell Type, and Brain Region—which reflects the original audience targeted by the database.

Other limiters provide few options. For example, Publication Types limits currently only to journal article, review, study, meta-analysis, letters/commentary, clinical trial, editorial, news, case report, and dataset, although these filter options are evolving with the addition of biomedical literature. The Organism limiter refers to the study population species only, not the infectious agents being studied. Lists of extracted key phrases from citations often do little to further a search in the way that assigned Medical Subject Headings (MeSH) terms often can in PubMed.

## SIMILAR TOOLS

Semantic Scholar aims to combat the information overload and lack of quality assessment that many researchers experience with Google Scholar’s keyword search. To accomplish this, developers are currently relying on preexisting indexing in PubMed and IEEE, along with annotations provided by medical subject experts. Articles without full-text links instead link to a digital object identifier (DOI), PubMed abstracts, or IEEE.

Like Google Scholar, Semantic Scholar combs the web for citations via an undefined algorithm, which favors precision and full-text access over recall. Unlike Google Scholar, it does not search behind paywalls. Thus, despite the developers’ stated emphasis on quality, Sematic Scholar’s inability to search licensed resources favors time, convenience, and access. Hence researchers searching Semantic Scholar in its current form cannot consider it a complete search of the background literature in their disciplines.

PubMed now incorporates many of the visualization features that Semantic Scholar explores in terms of citation graphs. While Semantic Scholar has the added ability to limit to methods, it does not offer a limiter comparable to PubMed’s study type.

Other artificial intelligence–enabled search engines exist. Some have compared Semantic Scholar to the Memex project from NASA and DARPA [6] that searches the deep web, though that project is not available to the public. It is also compared to Meta [7], now owned by the Chan-Zuckerburg Initiative. Meta was designed in 2010 with a greater emphasis on predicting future impact, and at the time of this writing, it is not yet available to the public [8]. Another artificial intelligence–enabled search engine with a business focus, AlphaSense, has been available by paid subscription since 2010 [9].

## CONCLUSION

Despite limitations, Semantic Scholar achieves its aim of visually representing the important elements of a paper (key people, data analysis, and graphical representation of citations) without requiring a great deal of reading. As an introductory database on computer science or neuroscience or one meant to rapidly display impact to diverse stakeholders, it has value. Transitioning to biomedical literature will take longer and require greater oversight by subject experts. While it works to overcome barriers imposed by publisher paywalls, Semantic Scholar’s greatest value may be its ability to visualize open access research.
